# Severe Respiratory Alkalosis in Acute Ischemic Stroke: A Rare Presentation

**DOI:** 10.7759/cureus.7747

**Published:** 2020-04-20

**Authors:** Vijayadershan Muppidi, Sashank Kolli, Vasuki Dandu, Samata Pathireddy, Sreenath Meegada

**Affiliations:** 1 Internal Medicine, Indiana University Health, Indianapolis, USA; 2 Internal Medicine/Pulmonary and Critical Care, Indiana University Ball Memorial Hospital, Muncie, USA; 3 Neurology, Baptist Health Medical Center, Little Rock, USA; 4 Internal Medicine, Deaconess Health System/Indiana University School of Medicine, Evansville, USA; 5 Internal Medicine, The University of Texas Health Science Center/Christus Good Shepherd Medical Center, Longview, USA

**Keywords:** acute cva, respiratory alkalosis, acute encephalopathy, cryptogenic stroke

## Abstract

Respiratory alkalosis is a rare but severe complication of acute ischemic stroke (AIS). In ischemic stroke, respiratory alkalosis results from hyperventilation due to the effect of stroke on the respiratory center. We report a case of a young male who presented with acute encephalopathy. Work-up revealed ischemic infarcts in the bilateral cerebellar and left posterior cerebral artery territory. Arterial blood gas (ABG) showed severe respiratory alkalosis with a pH of 7.72. Alkalosis resolved with mechanical ventilation. Such a high pH associated with AIS has not been reported in the medical literature so far. The index case highlights the severity of respiratory alkalosis that can be caused by an AIS. We conclude that early diagnosis and management of severe respiratory alkalosis is crucial for survival and recovery.

## Introduction

Stroke has a significant healthcare burden across the world. In 2009, one out of every 19 deaths in the United States was attributable to stroke [1]. According to the 2013 guidelines from the American Heart Association/American Stroke Association (AHA/ACC), acute ischemic stroke (AIS) is an episode of neurological dysfunction due to restriction of blood supply to a part of the central nervous system leading to the brain, spinal cord, retinal cell injury/death [2]. The cell injury usually happens in a defined vascular distribution and is recognized clinically, pathologically, or on imaging [2]. Clinical symptoms and the area of damage evident on imaging is dependent on the blood vessels involved. Common large arteries involved in ischemic stroke are anterior cerebral artery (ACA), middle cerebral artery (MCA), and posterior cerebral artery (PCA). The usual mechanisms of stroke are atherosclerotic and cardioembolic [3]. Stroke is classified as cryptogenic stroke if the cause is unknown or cannot be identified [4]. The lesions in brain due to stroke can cause hyperventilation leading to respiratory alkalosis [5]. Respiratory alkalosis is defined as a pH above 7.45 due to a pulmonary process [6]. We report a case of AIS that caused severe respiratory alkalosis with a pH above 7.7, one of the highest pH associated with ischemic stroke reported in the medical literature so far.

## Case presentation

A 37-year-old male with a history of migraine, chronic back pain, seizure disorder, anxiety, and remote lower back spinal fusion surgery was brought to the hospital for headache, slurred speech, lethargy, visual hallucinations, and inability to get up from the floor. Symptoms started a day prior to presentation. His wife noticed mild drooping of the right side of his mouth. His wife endorsed nausea and vomiting. Home medications included hydrocodone-acetaminophen 5-325 mg every six hours as needed, gabapentin (dose unknown), and alprazolam (dose unknown). He never smoked, had occasional alcohol, and no history of illicit drug use. He works at a desk job for family-owned businesses.

On presentation, he was afebrile, had blood pressure (BP) 150/80 mmHg, heart rate 53 beats/min, respiratory rate 18-26/min, and O2 saturation 93% on room air. BMI was 32.4 kg/m2. Glasgow Coma Scale was 14. Cardiac auscultation did not reveal a murmur. Neurological examination was significant for lethargy, intact motor and sensory systems and decreased reflexes in all four extremities. The patient was not cooperative to test cerebellar functions and gait. Initial laboratory work-up in the ER was unremarkable (Table 1).

**Table 1:** Labs at the time of admission. AST, aspartate aminotransferase; ALT, alanine transaminase; LDL, low-density lipoprotein; HDL, high-density lipoprotein

White blood cell count	10.6 k/cumm
Hemoglobin	14.9 gm/dL
Hematocrit	42.4%
Platelet count	333 k/cumm

Sodium	140 mmol/L
Potassium	4.2 mmol/L
Chloride	105 mmol/L
Bicarbonate	26 mmol/L
Blood urea nitrogen	13 mg/dL
Creatinine	0.89 mg/dL
Glucose	136 mg/dL
Anion Gap	9 mmol/L

Alkaline phosphatase	84 units/L
AST	34 units/L
ALT	39 units/L
Total bilirubin	0.3 mg/dL

Lipid panel (mg/dL)	Levels in index patient	Reference range
Total cholesterol	155	<= 200
Triglycerides	227	<= 150
LDL	78	=> 40
HDL	32	0-100

CT head did not reveal intracranial hemorrhage or mass. Arterial blood gas (ABG) was obtained and showed severe alkalosis with a pH of 7.72, with pCO2 <20 mmHg. Serum bicarbonate was 28 mmol/L (Table 2).

**Table 2:** ABGs prior to intubation and postintubation. ABG, arterial blood gas

ABG	Preintubation	Postintubation
pH	7.72	7.47
PCO2 (mmHg)	< 20	29
PO2 (mmHg)	117	131
HCO3 (mmol/L)	Incalculable	21
Base excess (mmol/L)	Incalculable	- 2
O2 saturation (%)	99	99

Work-up for acute encephalopathy was done (Table 3).

**Table 3:** Work-up for acute encephalopathy and other pertinent labs. UA, urinalysis; ANA, antinuclear antibody; VDRL, venereal disease research laboratory; TSH, thyroid stimulating hormone

Lab	Level in index patient	Reference range
UA	Negative for nitrite, leucocyte esterase, no wbc or bacteria	Negative
Urine drug screen	Positive for benzodiazepines, opiates, oxycodone	Negative
Ammonia (Mcmol/L)	102	6-47
Sed rate (mmol/L)	8	0-15
ANA titer	< 1:80	< 1:80
VDRL syphilis	Nonreactive	Nonreactive
Vitamin B12 (pg/mL)	334	0 - 1000
TSH (mcU/mL)	0.331	0.4-4.2
T4 (ng/dL)	0.8	0.6-1.5
Folate (ng/mL)	20.7	>= 5.9
Methylmalonic acid (Mcmol/L)	0.13	0-0.4

**Figure 1:**
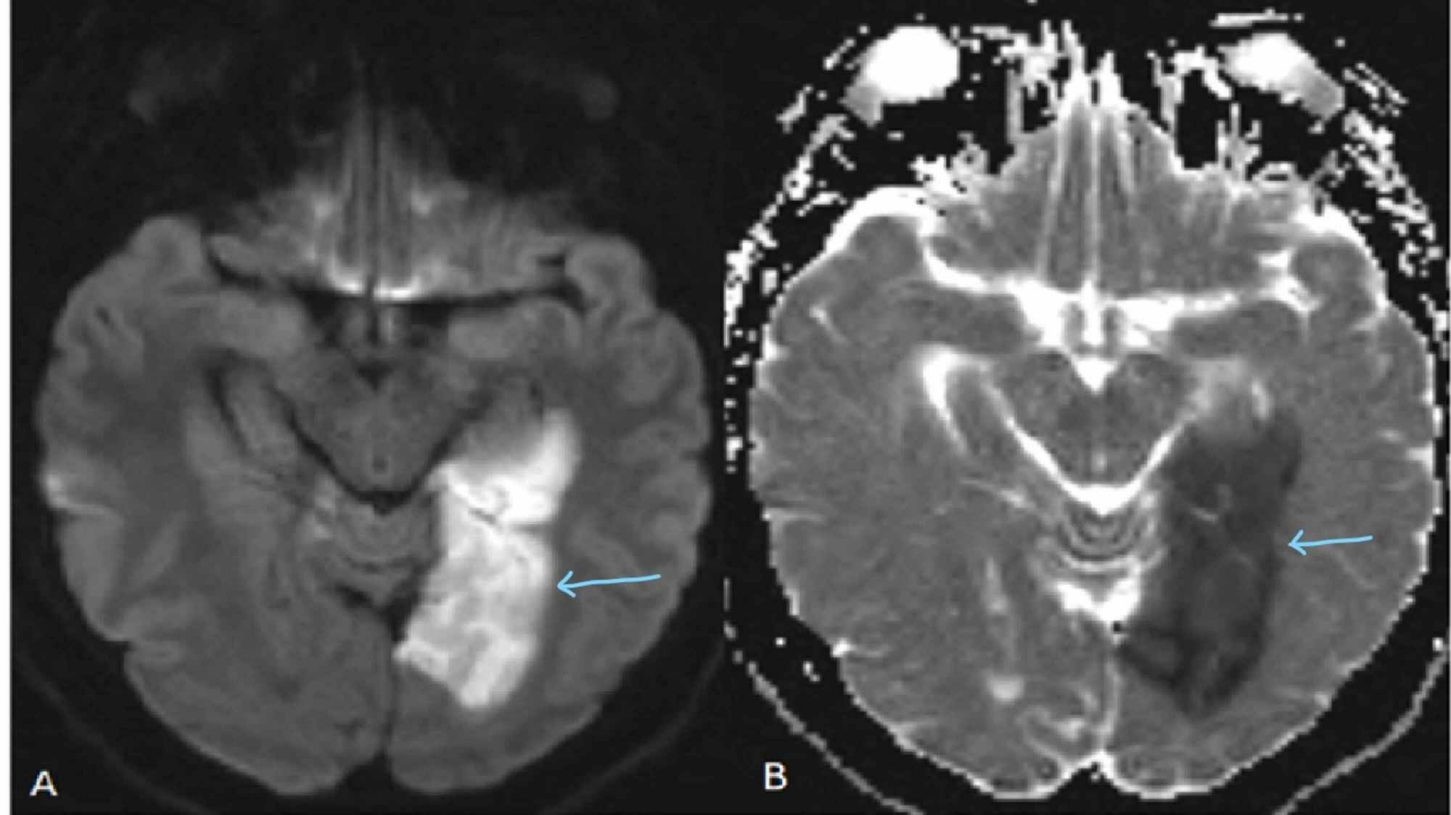
Diffusion weighted imaging (A) and corresponding ADC map (B) showing decreased diffusion in the left occipital lobe consistent with acute infarct (arrows pointing). ADC, apparent diffusion coefficient

**Figure 2:**
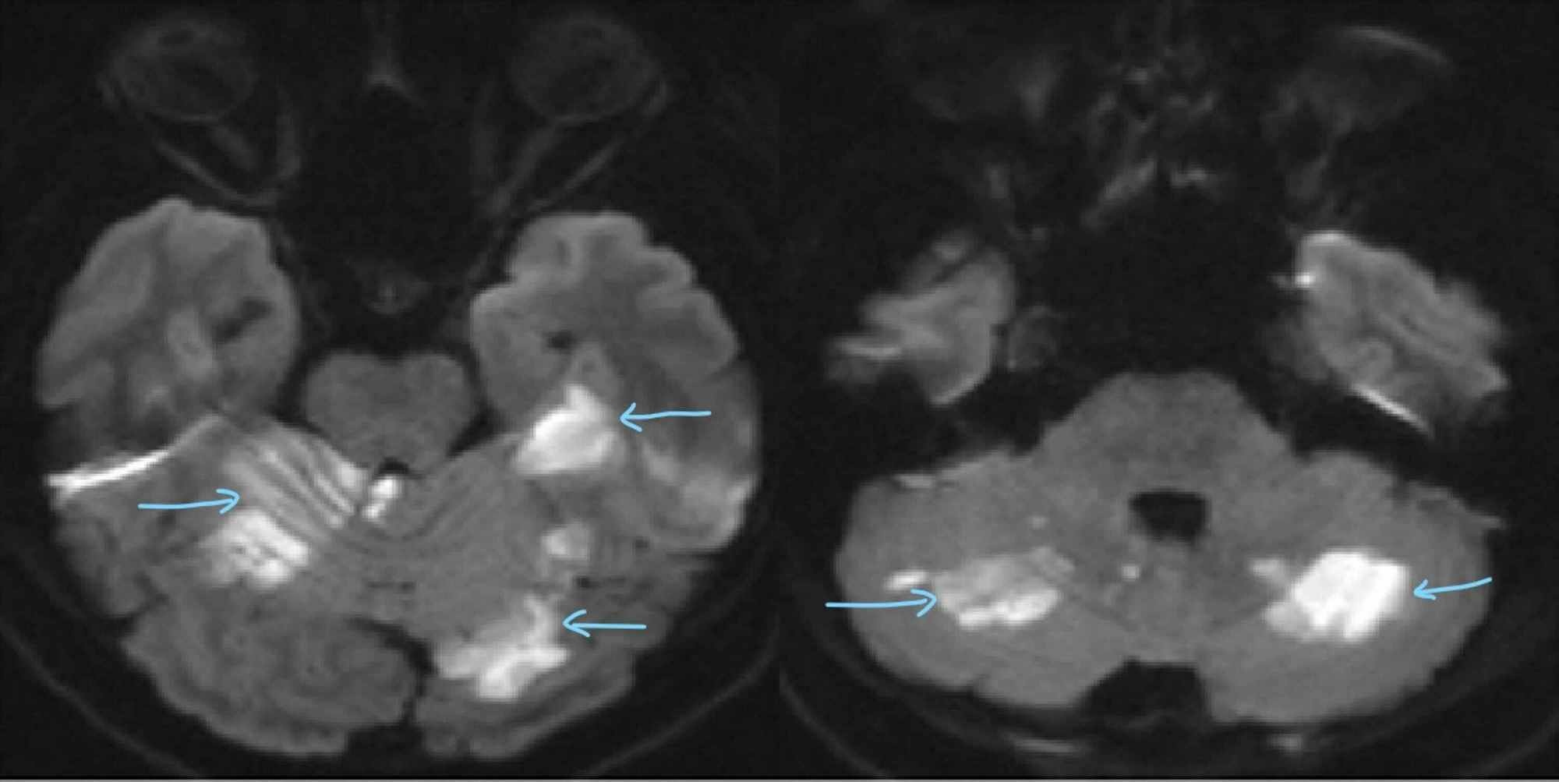
Diffusion weighted images show acute infarcts involving the left superior cerebellum (left side arrows) and bilateral inferior cerebellar hemispheres (right side arrows).

MRI brain diffusion weighted images showed acute ischemic infarcts in bilateral cerebellar hemispheres, superior vermis, and PCA distribution (Figures 1-2).

**Figure 3:**
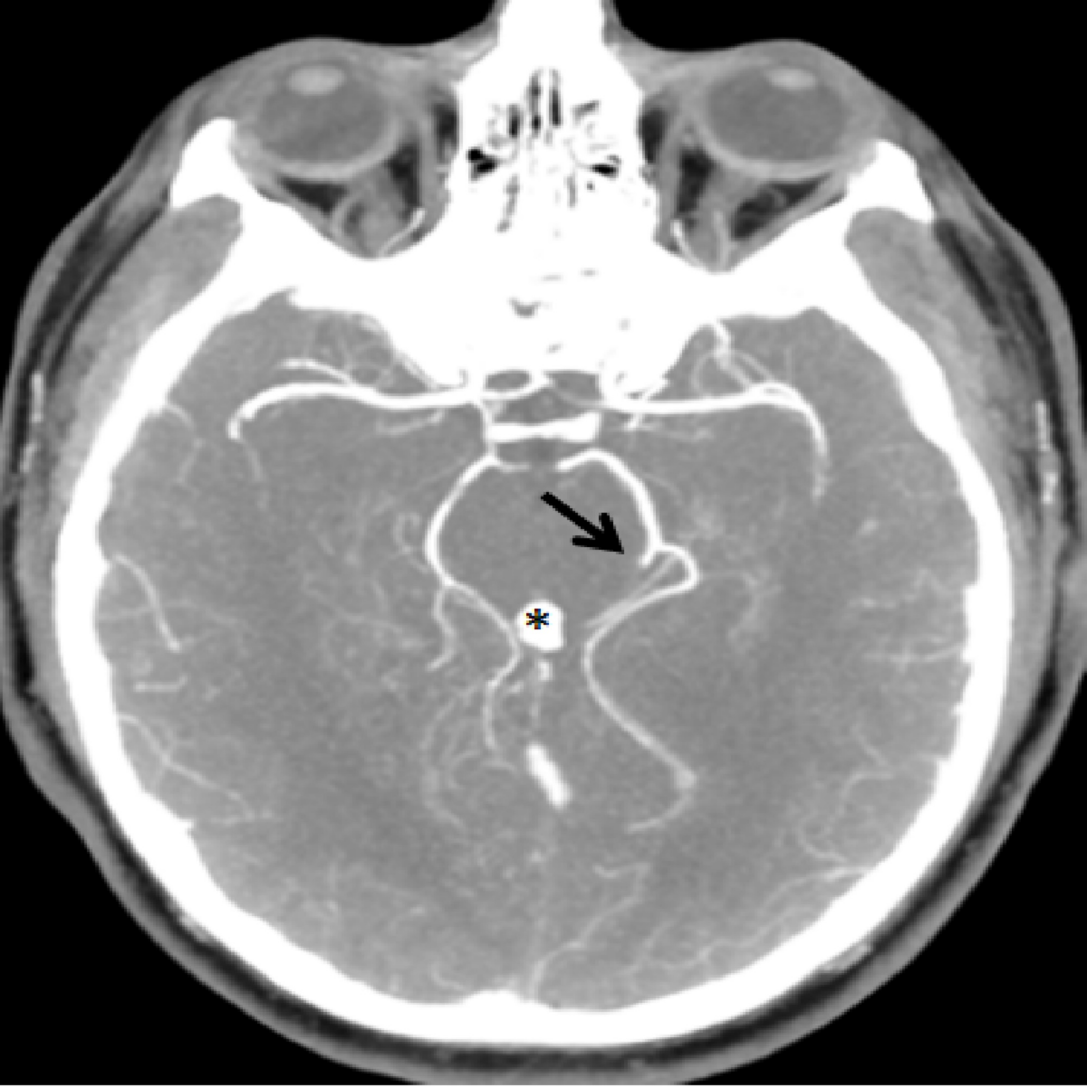
Maximum intensity projection of CT arteriography showing occlusion of a branch of the left posterior cerebral artery (arrow).

The CT angiogram of head and neck revealed a focal loss of gray-white differentiation involving the left temporal, occipital lobe in a left PCA distribution as well as a region in the left cerebellar hemisphere with no flow-limiting stenosis of the major arteries of the neck (Figure 3).

The patient was out of the therapeutic window for thrombolysis. The patient was started on aspirin, plavix, and high-intensity statin. Due to his altered mental status and severe respiratory alkalosis, he was transferred to ICU and intubated. Two hours postintubation, ABG showed improvement in the alkalosis (Table 2). The patient was extubated in two days. A transesophageal echocardiogram showed a patent foramen ovale (PFO). There was no evidence of venous thrombosis in lower extremities. No arrhythmias were seen on telemetry. He had some memory deficits but no motor or balance deficits by the time of discharge. The patient was discharged to a rehab facility in one week. He follows with the cardiology clinic at a tertiary center for the PFO closure.

## Discussion

The presentation of AIS depends on the blood vessel involved and the area of ischemia. Our patient presented with an ischemic stroke with infarction in the bilateral cerebellar and left PCA territory. This is a case of cryptogenic AIS as the etiology of cerebral ischemia is not known despite work-up [4]. He was found to have a PFO, without evidence of venous thrombosis. Currently, he is following with cardiology regarding PFO closure, which has been shown to have better outcomes than medical therapy in his age group [7-8].

The patient became encephalopathic due to the stroke leading to severe respiratory alkalosis. ABG revealed pH 7.72, PCO2 < 20 mmHg, and serum bicarbonate 28 mmol/L. The ABG is suggestive of acute primary and uncompensated respiratory alkalosis along with metabolic alkalosis. Respiratory alkalosis is the dominant acid base issue. The mild metabolic alkalosis was a result of vomiting. Vomiting causes metabolic alkalosis due to loss of hydrogen ion and hypovolemia [9]. But the dominant acid base abnormality in this case is primary respiratory alkalosis. Respiratory alkalosis is due to episodes of hyperventilation or sustained tachypnea/hyperpnea [10]. The index patient was hyperventilating at a rate of 18-26 at the time of presentation. This explains the respiratory alkalosis in the index case.

Based on the location of infarct on imaging, it is clear that the index patient suffered stroke in the left PCA and bilateral superior cerebellar artery territories. Ischemic stroke has been studied to cause respiratory alkalosis [10]. Various studies sought to explain the respiratory alkalosis in acute stroke. Primary central neurogenic hyperventilation has been suggested as a rare entity of hyperventilation without underlying pulmonary etiology and occurs despite alkalosis and low PCO2 [11]. The respiratory center has long known to reside predominantly in the brainstem, in pons and medulla [12]. Studies have been done recently to explore more about the respiratory centers in the nervous system [12]. Rowat et al. (2007) reported that respiratory control in the brain might be more spread out rather than reside in a single center. Furthermore, they also point out the possible role of humoral agents released from infarcted areas that influence respiratory pattern [13]. Through these studies, it is becoming more evident that respiratory and breathing patterns may not be related to one particular location or severity of stroke [14].

Respiratory pattern disturbances, including central periodic breathing (CPB)/Cheyne-Stokes Breathing, have been associated with acute stroke [15-16]. Cheyne-Stokes Breathing is a pattern of hypoventilation alternating with hyperventilation and has not been associated with any particular location of stroke [13-14]. Lee et al. noticed respiratory alkalosis in almost all stroke patients with Cheyne-Stokes Breathing pattern [16]. Kim et al. noticed that Cheyne-Stokes Respiration occurs irrespective of the location or severity of stroke [14]. Although, as per Rowat et al., Cheyne-Stokes Breathing is associated more with anterior circulation strokes [15].

Posterior circulation is divided into proximal (medulla and posterior inferior cerebellum), middle (pons and anterior inferior cerebellum), and distal (rostral brain stem, superior cerebellum, occipital and temporal lobes) [17]. The index patient had a stroke in the distal posterior circulation territory. Distal posterior circulation includes supply to rostral brainstem. Some brain stem involvement could have been possible and has been suggested by Anderson and Henrich in partial territory strokes [18].

There are a couple of salient but exciting features about our case. To our knowledge, such a high pH (> 7.7) associated with ischemic stroke has not been reported in the medical literature so far. There a few cases reported with a pH > 7.7 due to causes other than stroke. Also, we did not come across a respiratory alkalosis (plus mild metabolic alkalosis) with such a high pH as the cases with severe alkalosis previously reported are almost all due to metabolic alkalosis. Severe alkalosis causes metabolic disorders and several severe and detrimental complications to the patient's health [19]. A study by Sur and Shah found that patients with respiratory and metabolic alkalosis had a poor outcome, about 44.2% mortality [20]. In our case, early diagnosis and management of respiratory alkalosis resulted in the survival and recovery of the patient. He was not found to have any significant deficits during the post-recovery and follow-up period.

## Conclusions

This case report highlights the potential, rare, but severe complication of respiratory alkalosis with AIS. The patient was successfully managed and has survived. Clinicians should keep this in mind in patients with AIS presenting with severe acute encephalopathy. Early diagnosis and management are crucial in survival and recovery.
